# Effects of the Diet Inclusion of Common Vetch Hay Versus Alfalfa Hay on the Body Weight Gain, Nitrogen Utilization Efficiency, Energy Balance, and Enteric Methane Emissions of Crossbred Simmental Cattle

**DOI:** 10.3390/ani9110983

**Published:** 2019-11-18

**Authors:** Wuchen Du, Fujiang Hou, Atsushi Tsunekawa, Nobuyuki Kobayashi, Toshiyoshi Ichinohe, Fei Peng

**Affiliations:** 1 The United Graduate School of Agricultural Sciences, Tottori University, Tottori 680-8550, Japan; duwuchen1990@126.com; 2 State Key Laboratory of Grassland Agro-Ecosystems, Key Laboratory of Grassland Livestock Industry Innovation, Ministry of Agriculture, College of Pastoral Agriculture Science and Technology, Lanzhou University, Lanzhou 730020, China; 3 Arid Land Research Center, Tottori University, Tottori 680-0001, Japan; kobayashi.nobuyuki@alrc.tottori-u.ac.jp; 4 Faculty of Life and Environmental Science, Shimane University, Matsue 690-8504, Japan; toshi@life.shimane-u.ac.jp; 5 International Platform for Dryland Research and Education, Tottori University, Tottori 680-0001, Japan; pengfei@tottori-u.ac.jp; 6 Key Laboratory of Desert and Desertification, Northwest Institute of Eco-Environment and Resources, Chinese Academy of Sciences, Lanzhou 730000, China

**Keywords:** leguminous forage, digestibility, energy utilization efficiency, nitrogen metabolism, dryland

## Abstract

**Simple Summary:**

Nitrogen utilization efficiency and enteric methane emission from ruminants remain the primary concerns when developing ruminant feed globally. Nitrogen utilization efficiency is the ratio of retained nitrogen in body tissue to the total nitrogen intake, which is the main factor in the body weight gain of ruminants, and usually range from 15% to 40%. The methane emissions of ruminants are an inevitable by-product when feeds have been fermented in the rumen and represents a 2% to 12% loss of diet energy. The low nitrogen utilization of ruminants can damage air quality and lead to soil nitrification and acidification, whereas high methane emissions from ruminants can increase global warming. Our study investigated the effects of two kinds of legumes (alfalfa and common vetch) with different levels (20% vs. 40%) of total dry matter allowance on body weight gain, nutrient digestibility, nitrogen utilization efficiency, and enteric methane emissions for crossbred Simmental cattle. Our results suggested that nitrogen utilization efficiency and methane emissions are significantly affected by the legume species and proportions. These results could be beneficial for the development of regional or national ruminant feeding systems, thereby improving nitrogen utilization efficiency and reducing methane emissions.

**Abstract:**

A low nitrogen utilization efficiency (NUE, the ratio of retained N to N intake) and high methane (CH_4_) emissions of ruminants can lead to potentially high diet protein wastage and directly contribute to global warming. Diet manipulation is the most effective way to improve NUE or reduce CH_4_ emissions. This study investigated how replacing oat hay with alfalfa hay (AH) or common vetch hay (CVH) with different proportions (20% (20) and 40% (40) of the total dry matter (DM) allowance) affects the body weight gain (BWG), NUE, and CH_4_ emissions of crossbred Simmental cattle. The forage dry matter intake (DMI) and the total DMI of cattle fed on a CVH40 diet were significantly higher than the values for those fed on AH20 or AH40 diets (*p* < 0.05). There were no differences in the BWG for the four treatments observed, however, nutrient digestibility significantly decreased in the AH40 diet as compared with the AH20 diet (*p* < 0.05). The NUE was significantly lower in AH40 than in CVH20. The CH_4_ emissions were significantly lower for the CVH40 diet than with the AH20 diet (*p* < 0.05). Our findings suggest that a 20% AH and 40% CVH substitution for oat hay are the optimal proportions to maintain the BWG, NUE, nutrient digestibility, and reduce the CH_4_ emissions of crossbred Simmental cattle. Overall, CVH has a greater potential to reduce CH_4_ emissions than AH.

## 1. Introduction

The impacts of the low nitrogen utilization efficiency (NUE) and high enteric methane (CH_4_) emissions of beef cattle remain the primary concerns in the development of ruminant feeding systems [1]. A low NUE could contribute more ammonia emissions to the air and more manure N outputs to the soil [2], which could damage air quality [3] and lead to soil nitrification and acidification [2]. The enteric CH_4_ emissions from ruminants not only represent a loss of diet energy [4] but could also contribute to global warming [5]. The development of a diet that can improve the NUE and reduce enteric CH_4_ emissions is in demand and would be beneficial to both animal husbandry and in facing global environmental challenges [6,7].

Grass occupies an important role in the ruminant feeding system as it represents a low-cost and abundant source of dry matter (DM). However, grass only is not capable of sustaining the required levels of animal production due to its low feeding value [8]. Hence, the interest in supplementing legumes into a grass-based diet because they are rich in protein and energy [9]. Previous studies in sheep have shown that the intake of organic matter (OM) and crude protein (CP), as well as ruminal ammonia nitrogen (N) concentrations increased with a 3:1 grass/legume mixture diet as compared to a diet of only grass [10]; the total tract digestibility of CP and digestible CP was significantly higher in a 1:1 alfalfa/oat mixture diet than oat only hay diet [11]. Moreover, alfalfa (78%) and grass (22%) pastures could reduce energy loss through CH_4_ emissions of cows as compared to grass-only pastures [12]. However, these studies focused on diets where supplementation of legumes was the only factor considered. Few studies explained the effects of diets with different levels of legumes on feed intake, digestibility, and CH_4_ emissions. In another study, the inclusion of 30% common vetch hay (CVH) was more optimal in reducing CH_4_ emissions than 0%, 10%, and 20% CVH diets but significantly depressed digestibility as compared to a 20% CVH diet [13]. Recently, Kobayashi et al. [14,15] concluded that 8% to 14% alfalfa hay (AH) in the warm season and 8% to 21% in the cool season were optimal when considering body weight gain (BWG), metabolizable energy (ME) intake, and increased economic benefits of growing beef cattle on a corn- and straw-based diet. 

Alfalfa (*Medicago sativa* L.) is the mostly widely planted perennial legume crop in the world and has been studied for many years [12,14]. Common vetch (*Vicia sativa* L.), a multipurpose annual cereal legume for livestock feed [16], not only plays an important role in dryland mixed farming systems [16] for grazing [17] or cutting for hay [18] but also meets the structural forage deficit in winter, which is linked to the seasonality of other feed sources [9]. Previous studies have shown that CP, digestible OM intake, and in vitro OM digestibility are significantly higher with the oat and common vetch mixture diet than with the oat-only diet for cattle [18], and the growth performance of animals is significantly higher with common vetch supplementation than without [19]. However, until now, there has been no available information on whether common vetch could substitute alfalfa in the ruminant feeding system or whether optimal proportions of common vetch could replace alfalfa. Therefore, the objective of this study was to investigate how CVH versus AH affects BWG, N metabolism (i.e., N digestibility, ruminal ammonia-N, and blood urea N (BUN) concentrations), and CH_4_ emissions associated with ruminal fermentation parameters using two different proportions (20% (20) and 40% (40) of the total DM allowance) for growing crossbred Simmental cattle in dryland environments at similar CP and predicted ME levels.

## 2. Materials and Methods

The Animal Ethics Committee of Gansu Province, China, approved the experimental protocols (file No. 2010-1 and 2010-2). This study was conducted in Linze Grassland Agriculture Trial Station, Lanzhou University, Zhangye City, Gansu Province, China (latitude 39.24°N, longnitude 00.06°E, 1390 m a.s.l.), which is characterized as a typical temperature continental climate because its average annual precipitation is 121.5 mm and annual average temperature is 7.7 °C. In this study, the AH was second cut, and common vetch (*Vicia sativa* L.cv. Lanjian No. 3) was harvested at the flowering stage and restored as common vetch hay (CVH). Oat hay (OH; *Avena sativa* L.) was purchased from a forage company (Sanbao Agricultural Company, Zhangye, Gansu, China). The ingredients for the concentrate (maize, soybean meal, and wheat bran) were acquired from a local source. The chemical composition of the forage and ingredients of the concentrate are shown in Table 1.

### 2.1. Animals, Treatments, and Diets

The Animal Ethics Committee of Gansu Province, China, approved the experimental protocols. This experiment involved 16 crossbred male Simmental cattle (Simmental × local cattle) with initial body weights (BWs) of 216 ± 24.4 kg (mean ± standard deviation, 10 months of age) at the start of the experimental period. The experiment used a randomized block experimental design with a 2 × 2 factorial arrangement of diets. All 16 cattle were allocated to one of the 4 treatments. The forage to concentrate ratio was fixed (60:40, DM basis) for all diets. Diet treatments used two kinds of legumes (AH and CVH) and two different OH-to-AH/CVH ratios in the diet (40:20 or 20:40, DM basis), indicated as follows: 20% CVH and 40% OH (CVH20), 40% CVH and 20% OH (CVH40), 20% AH and 40% OH (AH20), and 40% AH and 20% OH (AH40). This experiment consisted of 2 feeding periods. Each period consisted of a 14 day diet adaptation in the cowshed and 32 day data collection period in the chambers.

The target BWG for each cattle was set at 1.5 kg/d. All experimental diets were formulated to provide sufficient ME and metabolizable protein (MP) to meet the target BWG for cattle according to the published estimation equations and values of the Agricultural and Food Research Council [20] and BW of cattle (measured every 8 days). The diet composition required to fulfill the ME and MP requirements was calculated based on the tabulated values of digestible energy and ruminal CP degradation parameters for OH, AH, and the concentrate ingredients established by the Chinese Feeding Standard for Beef Cattle [21]. The digestibility of ruminal CP and energy and ruminal degradation parameters for CVH were taken from Larbi et al. [16]. The CP, ME, and MP levels of all diets are shown in Table 2. Throughout this experimental period of 8 weeks, all cattle were given free access to water and 10 g/day of mineral mixture containing (minimum values in mg) manganese, 720; copper, 30; biotin, 0.05; folic acid, 0.4; vitamin B_1_, 50; vitamin B_2_, 2.5; vitamin B_6_, 0.5; and vitamin B_12_, 0.1. The daily mixed forage was divided into two equal parts and offered as separate meals twice a day (08:00 and 19:00). The mixed concentrate was fed once a day (14:00).

### 2.2. Chamber Description

The four indirect open-respiration calorimeter chambers used in the present study were equipped with a computer-controlled air-handling system with air conditioning units set to a temperature of 18 ± 1 °C and relative humidity of 60% ± 10%. The calorimeter chambers were built with double Perspex walls fitted in aluminum frames [22], with a total volume of approximately 18 m^3^ (4.2 m long, 1.95 m wide, and 2.2 m high). Each chamber was equipped with a gas flow meter (GFM57, Aalborg, Orangeburg, New York, NY, USA) at the outflow site for recording the total airflow and an engine to ensure a slight negative pressure within the chamber. All chambers were ventilated by suction pumps with a flow rate of 45 to 50 m^3^/h. The exhaust air was removed for volume, temperature, and humidity measurement and analysis in the bottom, middle, and upper areas, inside each chamber. The concentrations of CO_2_, CH_4_, and O_2_ in the air moving into and out of each chamber were measured every 16 min (the interval for each chamber) using a multigas analyzer (VA-3000, Horiba Ltd., Tokyo, Japan) in a general control room. The analyzer was calibrated using standard gases (O_2_-free N_2_ and a known quantity of CH_4_, CO_2_ and O_2_, Dalian Special Gases Co., Ltd., Liaoning, China) at the beginning of the gas exchange collection period in each experiment. The determined concentrations were in an absolute range of 0–500 μL/L for CH_4_, 0–2000 μL/L for CO_2_, and 0%–25% (*v*/*v*) for O_2_ (with linearity within this range). The recovery rate of CH_4_ was determined by comparing the CH_4_ release into the chamber with a given concentration as well as the CH_4_ concentration at the outlet. The gas recovery rate was approximately 100% ± 2% for all chambers, as highlighted recently by Gerrits et al. [23]. Each chamber was designed with a dedicated door, which was located next to the animal trough. The staff only opened the door to feed the animal immediately after the completion of each data collection in the chamber during the 3 day gas exchange data collection period. This minimized the effects of feeding activity (less than 1 min) on the gas concentrations inside. The CH_4_ emissions were expressed as the average CH_4_ emissions (g/day) from 3-day measurements divided by BWG, which was calculated from the BW change between moving in and moving out the chamber.

### 2.3. Energy Balance

ME intake (MEI) was calculated as the difference between GEI, excreted fecal energy (FE), and the sum of UE and CH_4_ energy (CH_4_-E) output. Retained energy (RE) was calculated using the equation MEI − heat production (HP). CH_4_-E was calculated from CH_4_ emissions (L/day) and the conversion coefficient (39.54 kJ/L; [24]). The CH_4_ emissions was converted to grams from the CH_4_ emissions (L/day) using the conversion coefficient (0.716 g/L, [24]). HP (kJ/day) was calculated with the following equation [24]:

$$\mathrm{HP}\;\left(\mathrm{kJ}/\mathrm{day}\right)\;=\;16.18\;\times O_{2}\;\mathrm{consumption}\;\left(L/\mathrm{day}\right)\;+\;5.02\;\times {\mathrm{CO}}_{2}\;\mathrm{production}\;\left(L/\mathrm{day}\right)\;\;-\;2.17\times \;{\mathrm{CH}}_{4}\;\mathrm{production}\;\left(L/\mathrm{day}\right)\;-\;5.99\;\times \;N\;\mathrm{excretion}\;\left(\mathrm{urinary}\;N,\;g/\mathrm{day}\right)$$

### 2.4. Sample Collection and Procedures

The amount of offered forage and concentrate and all leftovers was weighed daily throughout the experimental period to calculate the daily DM intake (DMI) for individual cattle. On day 15 of the experimental period, after the 14 day acclimation period for target feeds, one cattle was randomly selected from each diet group and moved to one of the four chambers for 8 days. On day 22, these cattle were moved to the individual pens in the cowshed, and another 4 cattle, randomly selected from the remaining cattle of the four diet groups, entered the chambers and left on day 30. This process continued until day 46 for the first feeding period, when all 16 cattle had completed 8 days of measurement. These acclimation and chamber measurements for metabolism and gas exchange were repeated for another 46 days with the 16 cattle randomly allocated to the four diets. The BWs of all cattle were measured in the morning with an empty stomach to calculate CH_4_ emissions, energy, and N balance based on the metabolic BW when exchanging cattle between the chambers and the cowshed. The BWG (kg/day) was calculated by the difference of the BW at the start and end time of each feeding period. During the 8 days of measurements in the chamber, the cattle were kept for acclimation for the first 2 days. We collected the digestibility data over the following 3 days and gas exchange data (O_2_ consumption, CH_4_, and CO_2_ emission) over the remaining 3 days. During the digestibility data collection period, the total weight of the daily excreted feces and urine was recorded. Feces, which were excreted onto a plastic mat placed under the cattle, were collected right after excretion with a shovel and placed into a plastic container (around 15 times a day but varied according to individuals) and weighed, mixed, and sampled once per day. A total of 10% of each feces sample was stored at −20 °C for later chemical analysis. All urine was collected once a day through a handmade urine bag into a bucket containing 200 mL 10% (*v*/*v*) H_2_SO_4_ to reduce ammonia loss. Acidified urine was checked for pH with a portable pH instrument (PHBJ-260, Shanghai INESA Scientific Instrument Co., Ltd., Shanghai, China). A total of 20% of the daily urine was removed with a 500 mL cylinder (deviation ± 5 mL) and stored at −20 °C for chemical analysis.

Rumen fluid samples were taken from each cattle 4 h post forage supply every morning using stomach tubing on the last 2 days of each feeding period after these cattle were moved to the cowshed. The collected samples were immediately measured for pH using a portable pH meter (PHBJ-260, Shanghai INESA Scientific Instrument Co., Ltd., Shanghai, China), strained through two layers of muslin (mesh size 1 mm^2^) and stored at −20 °C for volatile fatty acid (VFA) analysis. An additional 1 mL of strained rumen fluid was deproteinated by adding 0.2 mL metaphosphoric acid (215 g/L) and 0.1 mL internal standard (Crotonic acid), and the VFA concentrations were determined by a gas chromatograph (Trace1300, Thermo Ltd., Rodano Milan, Italy) fitted with a polar capillary column. The plasma urea N concentration was assumed to be equivalent to the BUN concentration in the serum, since urea diffuses freely into and out of blood cells [25]. 

### 2.5. Chemical Analysis

After the chamber measurement, the stored feces samples were thawed at room temperature for 12 h, and the feces samples from each cattle over the three days were then mixed. A portion of the thawed feces sample was used for the N measurement according to the Association of Official Analytical Chemists methods, method 976.05 [26]. The CP concentration was calculated by multiplying the N concentration by 6.25. The remaining samples were oven dried at 65 °C for 48 h and then ground to pass through a 1 mm screen. A portion of each dried sample, mixed forage, and concentrate samples were used to measure ash by combustion using a muffle furnace at 550 °C for 10 h (method 942.05 [26]). The organic matter (OM) content (g/kg DM) was calculated by 1000 ash content (g/kg DM). Another portion of each dried sample was finely ground to measure gross energy (GE), neutral detergent fibers (NDFs), and acid detergent fibers (ADFs). The GE of the samples was determined with an automatic isoperibol calorimeter (6400, PARR Instrument Company, Moline, IL, USA). The NDF and ADF concentrations were analyzed sequentially in an ANKOM 2000 fiber analyzer (ANKOM Technology, Fairport, NY, USA) following the protocol described by Van Soest [27]. The ash was included to facilitate the NDF and ADF analysis of all the forage, concentrate, and feces samples. The α-amylase for NDF analysis was used only for the concentrate samples. The urine samples from each cattle over the three days were also thawed at room temperature for 12 h and then mixed before determining their urinary energy (UE) by using an automatic isoperibol calorimeter (see above), and N was determined using Kjeldahl procedure described previously by the Association of Official Analytical Chemists [26]. For the UE measurement, 4 mL fully mixed urine was taken and absorbed by a filter paper of a known weight, and then the total energy of the filter paper with a urine sample was measured by an automatic isoperibol calorimeter after it became dry at room temperature. There were another 5 samples using the same filter paper (known weight) to be measured for energy content (MJ/kg), which was used to calculate the UE. The measurements of CP, NDF, and GE of the forage and concentrate of the diets also followed the above methods and instruments. The ether extract of the forage and concentrate was analyzed using an ANKOM XT15 Extractor (ANKOM Technology, Fairport, NY, USA).

### 2.6. Statistical Analysis

A one-way analysis of variance (ANOVA) and generalized linear model analysis were used to investigate the effects of legume species (LS), legume proportion (LP), and their interactions (LS × LP) on DMI, BWG, nutrient digestibility, energy balance, N metabolism, and energy/N utilization efficiency. Differences among the means were considered to be significant at a *p* ≤ 0.05 on the basis of Tukey’s test, unless otherwise stated. The statistical program used in the current study was IBM SPSS Statistics for Windows, version 19.0 (IBM Corp., Armonk, NY, USA).

## 3. Results

### 3.1. Feed Intake, Apparent Nutrient Digestibility, and BWG

**Figure 1:**
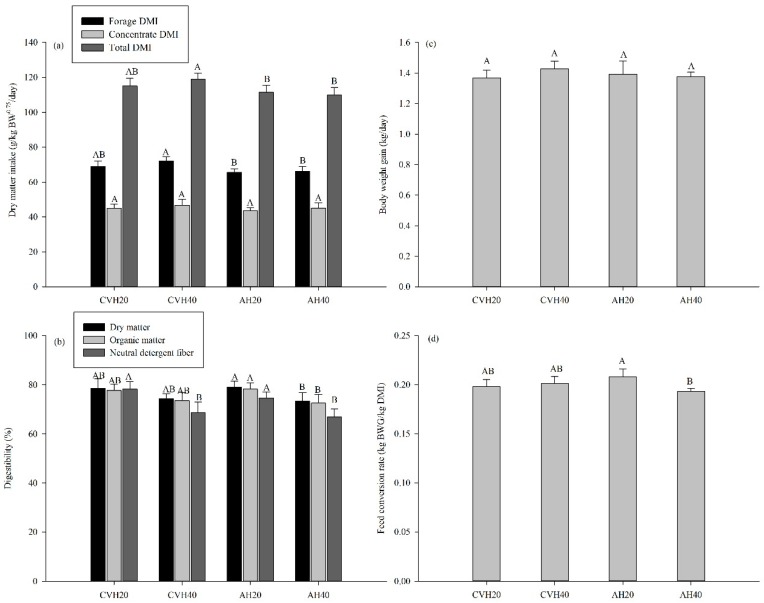
The dry matter intake (DMI) (**a**), digestibility (**b**), body weight gain (BWG) (**c**) and feed conversion rate (**d**) of cattle among the four diet groups. Values are presented as the mean ± standard deviation (SD). Uppercase letters only represent the difference among the four diet groups.

LS significantly influenced the forage DMI and total DMI (*p* < 0.05, Table 3). In detail, the forage DMI and total DMI of cattle were significantly higher when fed on a CVH40 diet than on AH20 and AH40 diets (*p* < 0.05, Figure 1a). However, no significant differences were found in the concentrate DMI under LS (*p* > 0.05, Table 3). In addition, there were no significant differences in the forage DMI, concentrate DMI, and total DMI of cattle under LP (*p* > 0.05, Table 3, Figure 1a).

LP significantly affected the nutrient digestibility of cattle, including the digestibility of DM, OM, NDF, and apparent N (*p* < 0.05, Table 3). Specifically, the digestibility of the DM, OM, and NDF of cattle when fed on an AH40 diet were significantly lower than those on an AH20 diet (*p* < 0.05, Figure 1b). In the CVH diet groups, only NDF digestibility was significantly lower in the CVH40 diet group than in the CVH20 diet group (*p* < 0.05, Figure 1b).

Both LS and LP did not significantly influence BWG and the feed conversion rate (FCR) of cattle (*p* > 0.05, Table 3), but the interaction between LS and LP had a significant effect on the FCR of cattle (*p* < 0.05, Table 3). In detail, the AH40 diet group had a significantly lower FCR than that in the AH20 diet group (*p* < 0.05, Figure 1d), whereas there was no difference between the CVH20 and CVH40 diet groups (*p* > 0.05, Figure 1d).

### 3.2. Enteric CH_4_ Emission, Energy Balance, and Energy Utilization Efficiency

**Figure 2:**
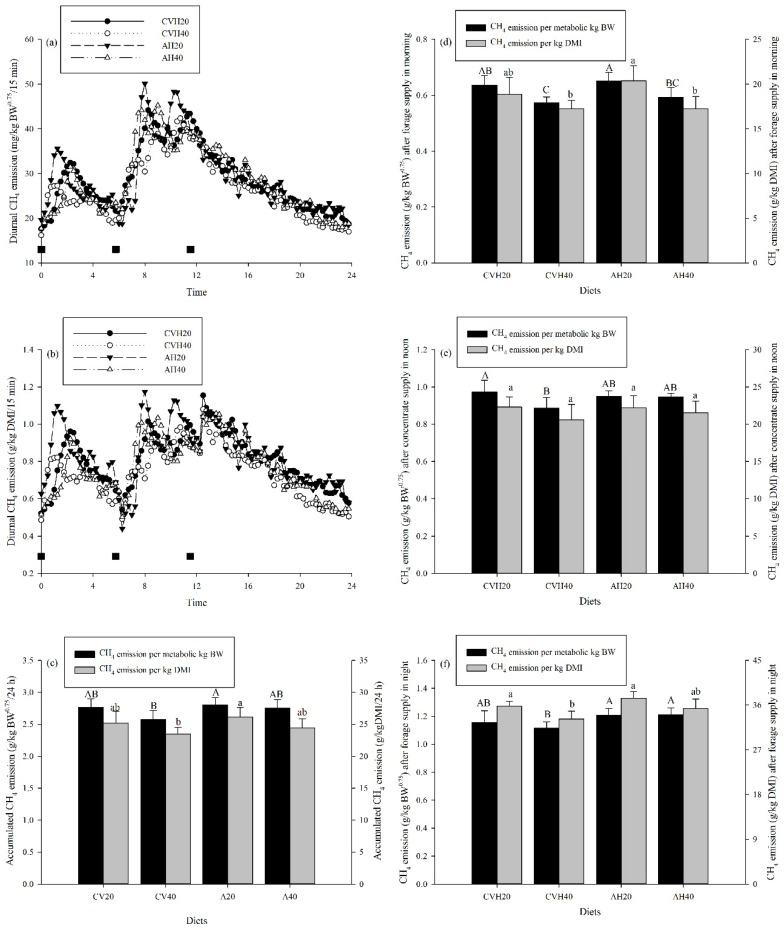
Diurnal CH_4_ emissions (**a**) and (**b**), accumulated CH_4_ emissions (**c**) after forage supply in the morning (**d**), after the concentrate supply in noon (**e**) and after forage supply in the night (**f**) for cattle among the four diet groups. Values are presented as the mean ± standard deviation (SD). Uppercase letters represent the differences among the four diet groups per kilogram of metabolic body weight, and lowercase letters represent the difference among the four diet groups per kilogram dry matter intake (DMI).

CH_4_ emissions, expressed on a milligram scale every 15 min per kilogram of metabolic BW and on a gram scale per kilogram DMI over 24 h post feeding, are shown in Figure 2a,b, respectively. There were intermittent peaks throughout the day and it was apparent that the peaks occurred a short time after feed supply. Moreover, the peaks of CH_4_ emissions (mg/kg BW^0.75^ or g/kg DMI) were relatively higher after the concentrate supply than after the forage supply (Figure 2a,b).

Both LS and LP could significantly affect CH_4_ emissions (g/kg BW^0.75^) over 24 h (*p* < 0.05, Table 3). Individually, the CVH diet groups had lower accumulated CH_4_ emissions (g/kg BW^0.75^) than the AH diet groups (Figure 2c), and the CVH40 and AH40 diet groups had relatively lower accumulated CH_4_ emissions (g/kg BW^0.75^) than the CVH20 and AH20 diet groups (Figure 2c). In addition, the accumulated CH_4_ emissions (g/kg BW^0.75^) were significantly lower in the CVH40 diet group than in the AH20 diet group (*p* < 0.05, Figure 2c). For CH_4_ emissions per kilogram DMI in a 24 h period, LP had a significant effect (*p* < 0.05, Table 3). In summary, the CVH40 diet group had significantly lower accumulated CH_4_ emission (g/kg DMI) than the AH20 diet group (*p* < 0.05, Figure 2c). 

Accumulated CH_4_ emissions per metabolic BW and per kilogram DMI after forage and concentrate supply in a 24 h period are shown in Figure 2d–f. Accumulated CH_4_ emissions (g/kg BW^0.75^) after forage supply in the morning were significantly lower in the CVH40 and AH40 diet groups than in the CVH20 and AH20 diet groups, respectively (*p* < 0.05, Figure 2d), and the CVH40 diet group had a significantly lower value than the AH20 diet group, regardless of forage supply in the morning or night (*p* < 0.05, Figure 2d,f). There was a similar trend between the accumulated CH_4_ emissions in grams per kilogram of metabolic BW and grams per kilogram DMI (Figure 2d). There were no significant differences in accumulated CH_4_ emissions (g/kg DMI) among the four diet groups (*p* > 0.05, Figure 2e) after concentrate supply, whereas the accumulated CH_4_ emissions (g/kg BW^0.75^) were significantly higher in the CVH20 diet group than in the CVH40 diet group after concentrate supply (*p* < 0.05, Figure 2e).

**Figure 3:**
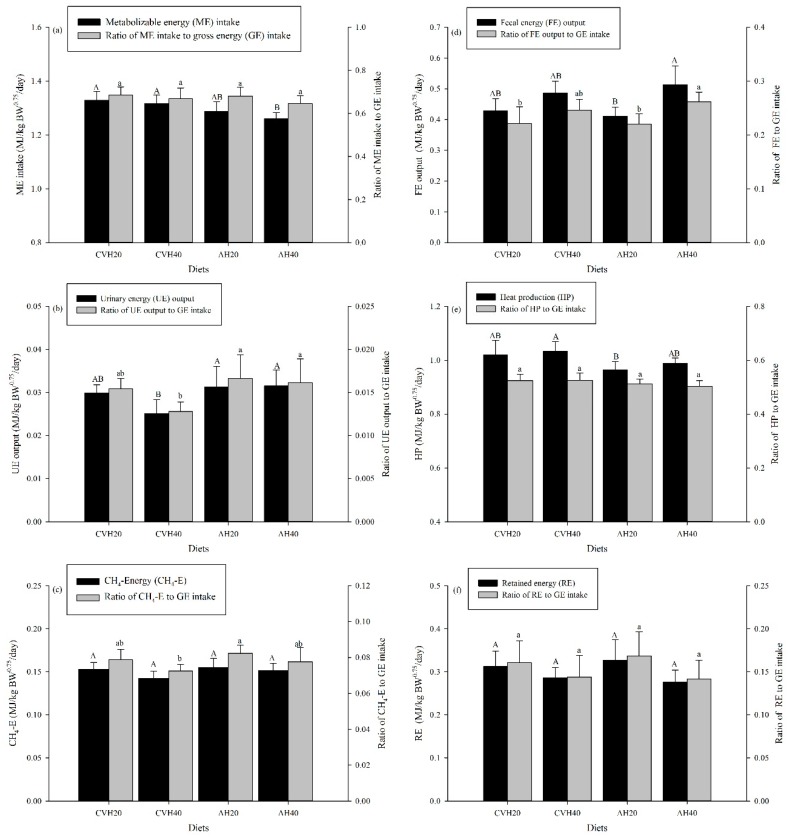
The energy balance and utilization efficiency of cattle among the four diet groups. Values are presented as the mean ± standard deviation (SD). Uppercase letters represent the differences among the four diet groups in energy balance, and lowercase letters represent the differences among the four diet groups in energy utilization. (**a**–**f**) represent ME intake, UE output, CH_4_-E, FE output, HP, and RE, respectively, as well as their proportion of GE intake.

LS only significantly affected MEI and HP (*p* < 0.05, Table 4). In particular, the CVH diet groups had higher MEI and HP than the AH diet groups (Figure 3a,e). Within the legume diet groups, LP only significantly influenced FE output (*p* < 0.05, Table 4). The CVH40 and AH40 diet groups had higher FE output than the CVH20 and AH20 diet groups (Figure 3d), whereas FE only significantly differed between the AH20 and AH40 diet groups (*p* < 0.05, Figure 3d). For energy utilization efficiency, LP only significantly influenced the ratio of FE to GEI (*p* < 0.05, Table 4). In detail, this ratio was significantly higher in the AH40 diet group than in the CVH20 and AH20 diet groups (*p* < 0.05, Figure 3d).

### 3.3. N Balance, N Metabolism, and NUE

**Figure 4:**
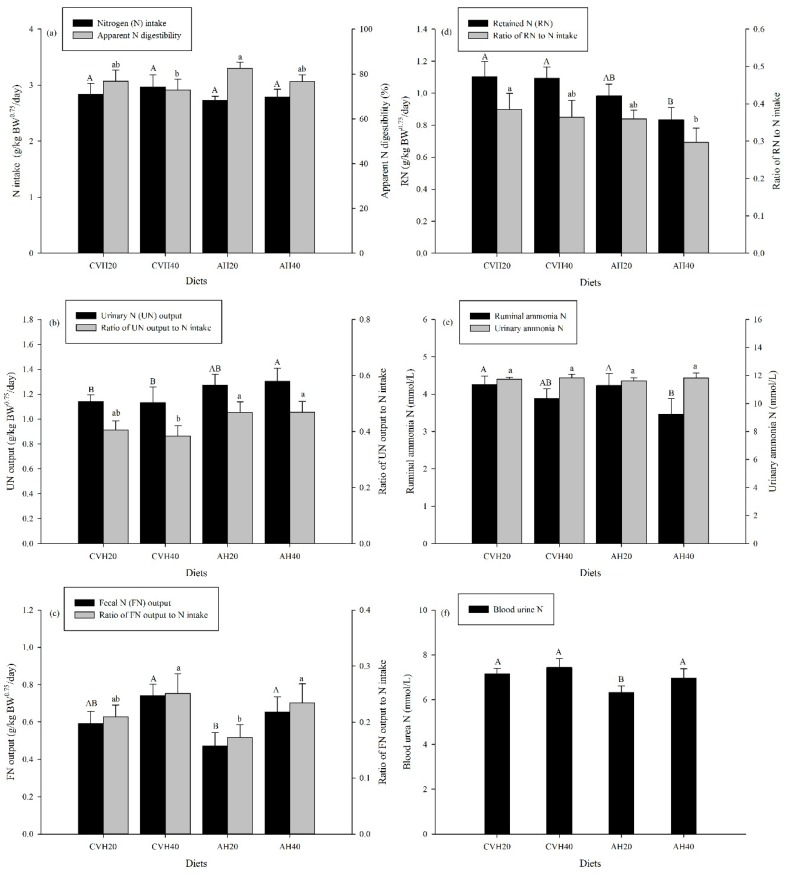
Nitrogen balance and utilization efficiency of cattle among the four diet groups. Values are presented as the mean ± standard deviation (SD). Uppercase letters represent the differences among the four diet groups in nitrogen balance and lowercase letters represent the differences among the four diet groups in nitrogen utilization. (**a**) represents N intake and apparent N digestibility; (**b**–**d**) represent UN output, FN output, and RN, respectively, as well as their proportion of N intake; (**e**) represents ruminal ammonia N and urinary ammonia N concentrations; (**f**) represents blood urea N.

LS did not affect the NI of cattle, but it significantly affected the FN, UN and RN outputs in N balance (*p* < 0.05, Table 4). Although the UN output of the CVH20 and CVH40 diet groups was significantly lower than that in the AH40 diet group (*p* < 0.05, Figure 4b), the CVH diet groups had a relatively higher FN output, especially between the CVH40 and AH20 diet groups (*p* < 0.05, Figure 4c). As a consequence, the RN of cattle in the CVH20 and CVH40 diet groups was significantly higher than that in the AH40 diet group (*p* < 0.05, Figure 4d). For the effect of LP on N balance, the CVH40 and AH40 diet groups had relatively higher FE outputs than the CVH20 and AH20 diet groups, but FE output only significantly differed between the AH20 and AH40 diet groups (*p* < 0.05, Table 4, Figure 4c).

LP could significantly influence ruminal ammonia-N concentration (*p* < 0.05, Table 4), which was significantly lower in the AH40 diet group than in the AH20 and CVH20 diet groups (*p* < 0.05, Figure 4e). Both LS and LP significantly affected BUN concentration (*p* < 0.05, Table 4), and AH20 had a significantly lower BUN than the AH20, CVH20, and CVH40 diet groups (*p* < 0.05, Figure 4f). No differences were found for the urinary ammonia-N concentration among the four diet groups (*p* > 0.05, Table 4).

LP significantly affected the ratio of FN to NI (*p* < 0.05, Table 4). The AH20 diet group had a significantly lower FN to NI than that in the AH40 and CVH40 diet groups (*p* < 0.05, Figure 4c). Moreover, LS significantly influenced the ratio of UN to NI (*p* < 0.05, Table 4) and the ratio of RN to NI (*p* < 0.05, Table 4). The CVH40 diet group had a significantly lower UN:NI than the AH20 and AH40 diet groups (*p* < 0.05, Figure 4b), and the CVH20 diet group had a significantly higher RN:NI than the AH40 diet group (*p* < 0.05, Figure 4d).

### 3.4. Ruminal Fermentation Parameters

The total VFA and pH of the ruminal fluid did not significantly differ among the four diet treatments (*p* > 0.05, Table 5). However, the molar proportion of acetate was significantly lower in the CVH40 and AH40 diet groups than in the CVH20 and AH20 diet groups, respectively (*p* < 0.05, Table 5). Additionally, the molar proportion of acetate was also significantly lower in the CVH diet groups than in the AH diet groups (*p* < 0.05, Table 5). The molar proportions of propionate in the CVH40 and AH40 diet groups were significantly higher than those in the CVH20 and AH20 diet groups, respectively (*p* < 0.05, Table 5). As a consequence, the ratio of acetate to propionate was significantly lower in the CVH40 and AH40 diet groups than in the CVH20 and AH20 diet groups (*p* < 0.05, Table 5).

## 4. Discussion

### 4.1. Feed Intake, Nutrient Digestibility, and BWG

In general, feed intake is restricted by the capacity of the rumen [22] and NDF content, which is a measure of cell wall content, and the digestibility of forage [28,29]. In this study, the higher forage DMI in the CVH diet groups than in the AH diet groups (*p* < 0.05, Table 3, Figure 1a) could be attributed to the lower NDF content in CVH (Table 1). This indicates that feeds equal in digestibility but different in NDF content would result in different intakes [22]. The similar DM digestibility (Figure 1b) in CVH20 and AH20, as well as CVH40 and AH40 (Figure 1b), along with the higher DMI in the CVH diet groups, confirm the above deduction. 

The digestibility of mixed feed is affected by the feed’s chemical composition [22]. For example, forage intake with increasing legume proportions could promote the passage rate of feedstuff in the rumen [12] because legumes have lower fiber content than grass, which reduces the retention time of forage in the rumen [22,30]. In this study, the lower NDF digestibility in diets with higher legume proportions than those in the lower legume proportion diets (Table 3, Figure 1b) confirms the above finding. Compared to grasses, highly lignified cell walls could decrease cell wall digestion in legumes and then decrease OM digestion in the rumen [31]. The lower OM digestibility in higher legume proportion diets as compared to those with lower proportions supports the previous finding (Figure 1b).

### 4.2. Enteric CH_4_ Emission and Ruminal Fermentation

There is a clear relationship between forage type, concentrate feed or starch intake, OM digestibility, and patterns of ruminal fermentation [4]. In this study, the lower CH_4_ emissions in the diets with higher proportions of legumes than in those with lower ones, regardless of the per kilogram metabolic BW or per kilogram DMI (*p* < 0.05, Table 3, Figure 2c), indicate that a diet with a higher proportion of legumes could decrease CH_4_ emissions. This is consistent with the findings of Lee et al. [32], who reported that increasing the percentage of white clover feed with perennial ryegrass could decrease CH_4_ emissions. This could be attributed to the polyphenolic compounds in legumes, such as condensed tannins, which have been previously shown to be negatively correlated with CH_4_ emissions [33]. For rumen fermentation, there is a negative relationship between CH_4_ emissions and propionate formation in the rumen, which could depress the activity of methanogens [4,5]. In this study, the lower ratios of acetate to propionate (Table 5) correspond to lower CH_4_ emissions after forage supply (Figure 2d) in diet groups with a higher proportion of legumes and this is consistent with the above finding. In addition, it has been reported that lipid supplementation could reduce CH_4_ emissions [34,35]. In this study, legumes have higher concentrations of crude fat (ether extract) than grasses (Table 2), which led to a higher crude fat concentration per unit of DM in the diets with higher proportions of legume than in those with lower proportions. The lower CH_4_ emissions in the CVH40 and AH40 diet groups than in the CVH20 and AH20 groups could also be explained in the same way. More importantly, feed intake is the single most important determinant of CH_4_ emissions [36]. In this study, there was no difference in the DMI between the diets with higher or lower proportions of legume (Figure 1a), but the lower OM digestibility (1b) of the diet with a higher proportion of legumes and a higher passage rate [12] leaves less time for microorganisms to ferment the feedstuff in the rumen [31]. Therefore, lower CH_4_ emissions were observed in CVH40 and AH40 than in CVH20 and AH20 (Figure 2c). 

In addition to the effects of LP on CH_4_ emissions, LS also affected CH_4_ emissions, especially on the basis of per kilogram metabolic BW (Table 3). The relatively lower CH_4_ emissions (g/kg BW^0.75^) of the CVH diet groups as compared to the AH diet groups at the same LP (Figure 2c) indicate that CVH has better potential to inhibit CH_4_ emissions than AH, especially after forage supply (Figure 2d,f). This might be due to the lower content of NDF and ADF in CVH than in AH, which is in agreement with Beauchemin [35], who reported that lower CH_4_ emissions for animals fed legumes could often be explained by the lower fiber content of their diets. Moreover, the production of propionate over acetate in the rumen could also reduce CH_4_ emissions in the rumen [35]. In this study, these changes in propionate and acetate also confirmed that the acetate molar proportion was lower in the CVH diet groups than in the AH diet groups (*p* < 0.05, Table 5), although there were no differences in the propionate molar proportion (Table 5). The ratio of acetate to propionate was around 4.77 in the CVH diet groups (Table 5), which was higher than the result of the study by Calabrò et al. [36], who reported a value of 2.28 for an OH and CVH mixture diet using an in vitro gas production technique. This could be attributed to differences that may exist in vivo and in vitro. For example, increases of in vivo rumen propionate concentrations were lower than those observed in vitro [37].

Increasing the inclusion of concentrates in the diet, especially starch content, was regarded as another way to reduce CH_4_ emissions [34,38]. In this study, the CVH40 and AH40 diet groups have a relatively higher proportion of maize than the CVH20 and AH20 diet groups (Table 2). As a consequence, lower CH_4_ emissions were observed in the CVH40 and AH40 diet groups, even though this value only significantly differed between the CVH20 and CVH40 diet groups per kilogram metabolic BW (Figure 2e). Moreover, CH_4_ emissions still tended to be lower in the higher proportion of maize diet groups per kilogram DMI, although this was not considered significant (Figure 2e). These results suggest that starch intake could suppress CH_4_ emissions, in accordance with previous studies [4,31].

### 4.3. Energy Balance

In ruminants, energy is lost in the form of feces, urine, and methane emissions [39]. In this study, FE output and the ratio of FE output to GE intake were greater in the CVH40 and AH40 diet groups than in the CVH20 and AH20 diet groups (Figure 3d). This could be explained by the higher passage rate of the diets with a higher proportion of legumes in the rumen [12], as well as decreased DM digestibility (Figure 1b) because the greater the DM excretion, the greater the FE loss. The ratio of UE output to GE intake, which in previous studies was found to range from 0.9% to 4.8% [39,40], is an indispensable element of energy loss, and high UE loss is more common when animals are fed a silage diet [41]. In this study, the mean 1.4% for the ratio of UE output to GE intake fell within the lower range of the quoted studies, but LP did not significantly influence UE output or the ratio of UE output to GE intake (Figure 3b). The relatively lower values of the ratio of CH_4_-E to GE intake in the CVH40 and AH40 diet groups could be explained by the lower OM digestibility (Figure 1b), which reduced the retention time of the feedstuff in the rumen.

ME intake, expressed as per kilogram metabolic BW, was higher in the CVH diet groups than in the AH diet groups (Table 3, Figure 3a), which could be attributed to the higher forage DMI in the CVH diet groups (Figure 1a) because CVH had a higher ME concentration (MEC) than AH (Table 1). However, no differences were found for the ratio of ME intake to GE intake among the four diet groups (Figure 3a). Nevertheless, the higher ratio of FE output to GE intake in the diet with a higher proportion of legumes (Table 4 and Figure 3d), which accounted for the largest part of the feed energy that could not be utilized by the animals [19], still tended to be lower in the CVH40 and AH40 than in the CVH20 and AH20 diet groups (Figure 3a). Additionally, the ratio of ME intake to GE intake for the crossbred Simmental cattle, in this study, was around 0.67, which was higher than the previously reported 0.47 for mature Simmental cows [42]. This could be attributed to a higher OM digestibility (averaged 75.4%) in this study ascompared to that (62.4%) in the previous study [42]. The higher ME intake (Table 3 and Figure 3a) alongside a lack of differences in RE (Figure 3f) in the CVH diet groups as compared to the AH diet groups could be attributed to an increased HP for the CVH diet groups than the AH diet groups (Table 4 and Figure 3e). This is consistent with the finding of Ferrell and Jenkins [43] that HP increased alongside increasing ME intake for crossbred beef cattle.

### 4.4. N Balance, N Metabolism, and N Utilization Efficiency (NUE)

N excretion in feces and urine represents a considerable N loss from ruminant husbandry [7,22]. In this study, N losses were affected by LS and LP, although LS and LP did not influence total N intake (Table 3). For example, the significantly higher FN output and the ratio of FN output to N intake corresponded with a higher proportion of legume (CVH40 vs. CVH20 and AH40 vs. AH20, Figure 4c). These were likely caused by the decreased nutrient digestibility (Figure 1b), as well as decreased apparent N digestibility (Figure 4a), which usually lead to more N being excreted in feces. As a result, the higher FN output (Figure 4c) (but no different UN output, Figure 4b) in the diet with a higher proportion of legumes (Figure 4b) led to a reduced RN in the lower proportion of the legume diets (*p* = 0.073, Table 3, and Figure 4d). The UN, FN, and RN outputs were influenced by LS (Table 3). The UN output in the CVH diet groups was lower than that in the AH diet groups (Figure 4b), whereas the FN output presented an opposite result (Figure 4c). The greater shift of N excretion from urine to feces in the CVH diet groups than in the AH diet groups was regarded as a way to reduce the impact of volatile N excretion on the environment [6] because urinary urea is rapidly hydrolyzed to ammonium and then converted to ammonia which is readily volatilized and lost from the farm system to the environment [44]. By contrast, fecal ammonia production is generally low due to the slow mineralization rates of organic nitrogenous compounds [3,7]. As a consequence, the RN in the CVH diet groups was higher than that in the AH diet groups (Figure 4d). Therefore, the CVH diet has a greater potential to reduce the effects of volatile N excretions on the environment than the AH diet.

Generally, high ruminal ammonia-N concentrations for optimal OM degradation will result in an increase in the loss of N through urine [45]. In this study, ammonia-N concentrations in the rumen tended to be lower with a higher proportion of legumes, especially in AH diets (Figure 4e). This difference could possibly be due to the relatively higher passage rate of feedstuff in the rumen with increasing legume proportions [12], thereby, yielding a lower OM digestibility (Figure 1b) and ammonia-N concentrations in the diets with higher proportions of legumes than in the diets with lower proportions of legumes.

In addition, BUN levels reflected the protein status of cattle and positively corresponded with changes in the ammonia-N concentration in rumen fluid [46]. In this study, BUN tended to be higher in the diets with a higher proportion of legumes (Figure 4f), which was inconsistent with ruminal ammonia-N concentrations (Figure 4e). This might be attributable to the lowest pH in AH40 (Table 5), which depressed the transport of ammonia across the rumen wall. Studies have shown that the permeability of the rumen wall for ammonia is pH dependent, and has a positive correlation with pH [47]. Additionally, although the ruminal ammonia-N concentration tended to be lower in the diets with higher proportions of legumes than in the diets with lower proportions of legumes, there was no reduction in BWG (Figure 1c). This suggests that adequate ruminal available N was provided from the diet to maximize microbial fermentation in the rumen under a ruminal ammonia-N concentration of around 4.0 mmol/L.

## 5. Conclusions

The results of this study suggest the following: (1) a higher proportion of legumes in the diet could reduce CH_4_ emissions and minimize the impact of volatile N excretion to the environment; (2) increasing legume proportions in the diet could reduce nutrient digestibility, whereas the degree of reduction differs between common vetch hay and alfalfa hay; and (3) common vetch hay has great potential to minimize the negative effects of CH_4_ emissions and N excretion into the environment. Therefore, an opportunity for strategic feeding exists by using alfalfa hay (20%) and common vetch hay (40%) to reduce the direct impact of volatile N excretion and CH_4_ emissions on the environment while maintaining BWG, as well as nutrient digestibility for crossbred Simmental cattle, in dryland environments.

## Figures and Tables

**Table 1:** Chemical composition of alfalfa hay, oat hay, common vetch hay, and ingredients of the concentrate used in the experimental diets.

Item ^†^	Alfalfa Hay	Oat Hay	Common Vetch Hay	Soybean Meal	Wheat Bran	Maize
OM, g/kg DM	905	942	918	935	931	983
CP, g/kg DM	168	60	177	465	182	83
NDF, g/kg DM	458	559	413	166	454	100
ADF, g/kg DM	347	407	302	102	186	20
Ether extract, g/kg DM	22	18	23	26	55	44
GE, MJ/kg DM	17.9	16.8	17.7	19.6	19.4	18.5
MEC ^§^, MJ/kg DM	8.7	9.0	9.5	13.0	10.9	13.4
MPC ^¶^, g/kg DM	62	68	71	87	73	90

^†^ OM, organic matter; CP, crude protein; NDF, neutral detergent fiber; ADF, acid detergent fiber; GE, gross energy; MEC, metabolizable energy concentration; MPC, metabolizable protein concentration; ^§, ¶^ They were calculated by the Agricultural and Food Research Council (1993) and the Chinese Feeding Standard for Beef Cattle (2004), see details in Methods and Materials.

**Table 2:** Composition of the feed ingredients and the target metabolizable energy concentration and metabolizable protein concentration of all diets.

Feed Formula	Experimental Diet ^†^			
	CVH20	CVH40	AH20	AH40
Forage				
Leguminous forage (g/kg DM)	200	400	200	400
Oat hay (g/kg DM)	400	200	400	200
Concentrate				
Maize (g/kg DM)	30	80	48	120
Soybean meal (g/kg DM)	92	25	107	56
Wheat bran (g/kg DM)	278	295	245	224
Nutrient value ^‡^				
CP (g/kg DM)	156.3	156.4	156.4	156.4
MEC ^§^ (MJ/kg DM)	10.05	10.05	10.05	10.05
MPC ^¶^ (g/kg DM)	102.9	94.6	106.1	101.4

^†^ CVH20, 20% common vetch + 40% oat hay; CVH40, 40% common vetch + 20% oat hay; AH20, 20% alfalfa + 40% oat hay; AH40, 40% alfalfa + 20% oat hay. ^‡^ CP, crude protein, MEC, metabolizable energy concentration, MPC, metabolizable protein concentration. ^§,¶^ These values were calculated by the Agricultural and Food Research Council (1993) and the Chinese Feeding Standard for Beef Cattle (2004); see details in Methods and Materials.

**Table 3:** A general linear model analysis of legume species (LS), legume proportion (LP), and their interaction effect on feed intake, digestibility, growth performance, and CH_4_ emissions (n = 8).

Item ^†^	LS ^‡^	LP ^‡^	LS × LP ^‡^
Dry matter intake (DMI)			
Forage DMI (g/kg BW^0.75^/day)	5.783 ^*^	0.932	0.498
Concentrate DMI (g/kg BW^0.75^/day)	1.108	1.189	0.001
Total DMI (g/kg BW^0.75^/day)	5.207 ^*^	0.109	0.598
Digestibility			
DM digestibility (%)	0.215	5.671 ^*^	1.303
OM digestibility (%)	0.306	6.744 ^*^	1.582
NDF digestibility (%)	1.177	18.476 ^***^	0.001
Apparent N digestibility (%)	5.515 ^*^	5.949 ^*^	0.265
Growth performance			
BWG (kg/day)	0.205	0.403	1.389
FCR (kg BWG/kg DMI)	0.077	2.515	5.796 ^*^
CH_4_ emissions			
CH_4_ emissions (g/kg BW^0.75^/24 h)	5.907 ^*^	7.056 ^*^	0.815
CH_4_ emissions (g/kg DMI/24 h)	1.698	5.604 ^*^	0.000

^†^ DMI, dry matter intake; DM, dry matter; OM, organic matter; NDF, neutral detergent fiber; BWG, body weight gain; FCR, feed conversion ratio (ratio of BWG divided by the total DMI). ^‡^ values are the F value, ^*^ *p* < 0.05, and ^***^ *p* < 0.001.

**Table 4:** A general linear model analysis of legume species (LS), legume proportion (LP) and their interaction effects on energy balance/nitrogen balance and energy/nitrogen utilization efficiency (n = 8).

Item ^†^	LS ^‡^	LP ^‡^	LS × LP ^‡^
Energy balance			
GE intake (MJ/kg BW^0.75^/day)	1.302	2.783	0.126
ME intake (MJ/kg BW^0.75^/day)	6.749 ^*^	1.132	0.127
FE output (MJ/kg BW^0.75^/day)	0.042	13.739 ^**^	1.054
UE output (MJ/kg BW^0.75^/day)	4.675	1.584	1.992
CH_4_-E (MJ/kg BW^0.75^/day)	1.604	2.225	0.684
HP (MJ/kg BW^0.75^/day)	6.208 ^**^	1.198	0.170
RE (MJ/kg BW^0.75^/day)	0.012	4.758	0.469
Energy utilization efficiency			
Ratio of ME intake to GE intake (MJ/MJ)	0.436	1.589	0.224
Ratio of FE output to GE intake (MJ/MJ)	0.392	8.630 ^*^	0.504
Ratio of UE output to GE intake (MJ/MJ)	4.647	2.254	1.025
Ratio of HP to GE intake (MJ/MJ)	2.189	0.148	0.171
Ratio of CH_4_-E to GE intake (MJ/MJ)	2.332	3.644	0.066
Ratio of RE to GE intake (MJ/MJ)	0.051	2.993	0.178
Nitrogen balance			
N intake (g/kg BW^0.75^/day)	2.956	1.317	0.168
FN output (g/kg BW^0.75^/day)	8.792 ^*^	21.653 ^***^	0.207
UN output (g/kg BW^0.75^/day)	9.602 ^**^	0.046	0.176
RN (g/kg BW^0.75^/day)	21.681 ^***^	3.876	3.038
N metabolism			
Ruminal ammonia N (mmol/L)	2.044	12.989 ^**^	1.685
Blood urea N (mmol/L)	14.243 ^**^	6.884 ^*^	0.970
Urinary ammonia N (mmol/L)	0.241	1.420	0.140
Nitrogen utilization efficiency			
Ratio of FN output to N intake (g/g)	3.464	12.862 ^**^	0.459
Ratio of UN output to N intake (g/g)	16.116 ^**^	0.311	0.398
Ratio of RN to N intake (g/g)	5.992 ^*^	4.759	1.252

^†^ GE, gross energy; ME, metabolizable energy; FE, fecal energy; UE, urinary energy; CH_4_-E, methane energy; HP, heat production; RE, retained energy; N intake, nitrogen intake; FN, fecal N; UN, urinary N; RN, retained N. ^‡^ Values are the F value, ^*^ *p* < 0.05, ^**^ *p* < 0.01, and ^***^ *p* < 0.001.

**Table 5:** Effects of different diets on the ruminal fermentation parameters in Simmental crossbred cattle.

Item	Experimental Diet ^†^				Variance Analysis ^‡^		
	CVH20	CVH40	AH20	AH40	LS	LP	LS × LS
Total VFA, mmol/L	75.4 ± 6.73	72.5 ± 7.22	77.8 ± 3.32	75.7 ± 9.98	0.536	0.423	0.011
pH	6.07 ± 0.16	6.12 ± 0.25	6.05 ± 0.08	6.01 ± 0.06	0.686	0.009	0.293
Molar proportions (mol/100 mol)							
Acetate	72.3 ± 1.24	70.8 ± 0.56	73.8 ± 0.64	72.7 ± 1.13	11.967 ^**^	6.503 ^*^	0.122
Propionate	14.4 ± 0.24	15.7 ± 1.08	13.9 ± 0.76	15.2 ± 0.75	1.382	10.576 ^**^	0.007
Butyrate	10.2 ± 1.11	10.2 ± 1.44	9.2 ± 0.72	8.9 ± 0.46	4.747	0.072	0.042
Iso-butyrate	1.1 ± 0.06	1.2 ± 0.19	1.1 ± 0.20	1.1 ± 0.15	0.173	0.640	0.539
Valerate	0.7 ± 0.12	0.6 ± 0.08	0.6 ± 0.18	0.6 ± 0.05	1.444	1.950	0.544
Iso-valerate	1.23 ± 0.09	1.44 ± 0.24	1.32 ± 0.23	1.37 ± 0.18	0.003	1.780	0.679
Acetate/propionate ratio	5.01 ± 0.11	4.53 ± 0.30	5.32 ± 0.30	4.78 ± 0.31	3.987	11.522 ^**^	0.036

^†^ CVH20, 20% common vetch + 40% oat hay; CVH40, 40% common vetch + 20% oat hay; AH20, 20% alfalfa + 40% oat hay; AH40, 40% alfalfa + 20% oat hay. ^‡^ Values are presented as the mean ± standard deviation (SD); LS, legume species; LP, legume proportion; LS × LP, interaction between LS and LP. Values are the F value; ^*^ *p* < 0.05; ^**^ *p* < 0.01.
